# Effects of Aquatic-Based Resistance, Balance, and Proprioceptive Training on Ankle-Foot Malalignments in Postmenopausal Obese Women

**DOI:** 10.7759/cureus.87571

**Published:** 2025-07-08

**Authors:** Durva Hande, Sandeep Shinde, Akshanda Dhumale, Harshal Y Kale

**Affiliations:** 1 Department of Musculoskeletal Sciences, Krishna College of Physiotherapy, Krishna Vishwa Vidyapeeth (Deemed to be University), Karad, IND; 2 Department of Physiotherapy, Krishna College of Physiotherapy, Krishna Vishwa Vidyapeeth (Deemed to be University), Karad, IND; 3 Department of Critical Care Medicine, Krishna Vishwa Vidyapeeth (Deemed to be University), Karad, IND

**Keywords:** aquatic therapy, balance, malalignments, obese, postmenopause, proprioception

## Abstract

Background: Aquatic therapy has become a valuable rehabilitation method due to water's unique properties, including buoyancy, resistance, hydrostatic pressure, and temperature regulation. The study focuses on musculoskeletal changes associated with menopause, particularly the alterations in muscle strength and functional performance observed in postmenopausal women. It aimed to compare the outcomes of aquatic exercise programs with those of land-based exercises in terms of resistance training, balance, and proprioception.

Objectives: This study aimed to determine the impact of aquatic resistance training on improving muscular strength, balance, and alignment in the lower extremities in postmenopausal obese women.

Methods: This study included a total of 146 participants, who were randomly assigned to two groups: Group A (Control) and Group B (Intervention), with 73 participants in each group initially. Following the exclusion of three participants, the final sample consisted of 71 participants in Group A and 72 in Group B. Over six weeks, participants underwent either land-based or aquatic-based exercise protocols, depending on group allocation. Outcome measures included the Numerical Rating Scale for pain, manual muscle testing (MMT) for strength, and the Star Excursion Balance Test (SEBT) for assessing balance and proprioception.

Results: The findings of this study demonstrated that aquatic-based resistance, balance, and proprioceptive training had a significantly greater impact on correcting ankle-foot malalignments in obese postmenopausal women compared to land-based exercises. Participants in the intervention group (Group B) showed notable improvements across multiple assessment parameters, including the Numerical Pain Rating scale, MMT, and SEBT. The statistical analysis revealed highly significant results (p < 0.0001), indicating that aquatic training was more effective in enhancing strength, balance, and foot alignment than traditional land-based exercises.

Conclusion: The study found that a six-week aquatic training program helped alleviate pain and improved the overall ankle-foot complex performance (strength, balance, and proprioception) in postmenopausal obese women.

## Introduction

Aquatic therapy has become a valuable rehabilitation method due to water's unique properties, including buoyancy, resistance, hydrostatic pressure, and temperature regulation. These attributes allow therapists to customize exercises by varying water depth and using buoyant tools like collars, rings, and kickboards. Patients can perform movements in different positions, standing, sitting, or lying down, while reducing joint stress and enhancing comfort through weightlessness. The water’s resistance naturally strengthens muscles, making aquatic therapy effective for pediatric, orthopedic, neurological, and cardiopulmonary rehabilitation [1,2]. Menopause has an overall negative impact on musculoskeletal health. It is linked to osteoporosis, osteoarthritis, and sarcopenia. Osteoporosis and accompanying fractures, as well as the pain and locomotor handicap that accompany them, have an impact on postmenopausal women's quality of life and lifespan [3]. As women age, the natural progression of aging, coupled with the onset of menopause, brings about profound changes in body composition. These changes often include an increase in body weight and fat accumulation, accompanied by a decline in muscle mass, strength, balance, aerobic capacity, mobility, and flexibility. Additionally, bone density tends to decrease, heightening the risk of osteoporosis and fractures. Such physical alterations gradually diminish fundamental human abilities, such as performing everyday tasks and maintaining independence. Over time, this decline significantly impacts the overall quality of life, as it compromises both physical functionality and overall well-being [4]. The prevalence of overweight and obesity in menopausal women necessitates heightened awareness and a comprehensive, multidisciplinary approach to managing their health. These conditions, often exacerbated by the hormonal and metabolic changes associated with menopause, significantly increase the risk of developing chronic diseases and other health complications. Muscle strength declines with age and may be closely linked to menopausal status, significantly affecting functional performance and quality of life in postmenopausal women. While overall muscle strength is essential, the reduction in power output is particularly critical, as it directly influences muscle function and mobility. This decline increases the risk of falls and fractures, posing a significant health concern. Postmenopausal women often face challenges maintaining muscle power due to hormonal changes, which further impact their physical stability and ability to perform daily activities [5,6]. There has been literature on the effects of water therapy in people with chronic conditions, focusing mostly on musculoskeletal ailments, heart disease, diabetes mellitus, multiple sclerosis, and Parkinson's disease, showing the best improvements after participating in aquatic programs [7]. The findings suggest that aquatic therapy, incorporating both aerobic and resistance exercises, serves as an effective alternative training method for enhancing neuromuscular function and overall fitness performance, particularly in healthy elderly women. This form of therapy has been shown to significantly improve various physical health parameters, including cardiorespiratory fitness, muscular endurance, strength, and balance. Additionally, it positively impacts certain aspects of body composition, making it a valuable training approach for active young adult women as well. By leveraging the unique properties of water, such as reduced joint impact and increased resistance, aquatic therapy offers a safe and effective way to promote physical health and functional abilities across different age groups [8,9]. There has been research on elderly women in which a water-based exercise and self-management program led to significant improvements in balance and overall quality of life for community-dwelling women aged 65 and older diagnosed with osteopenia or osteoporosis. These findings highlight the effectiveness of aquatic interventions in enhancing physical stability and well-being in older women with compromised bone health [10]. A similar research was carried out, which pertained to resistance, balance, and proprioceptive training in knee osteoarthritis (KOA) patients, where aquatic therapy was proven to be effective than land-based one [11]. Furthermore, there is an absence of standardized aquatic exercise programs designed to target individuals with these malalignments. Aquatic therapy is recognized for its potential benefits, including reduced joint stress, enhanced mobility, and improved balance; however, its specific impact on ankle-foot malalignments in this population remains underexplored. Understanding the underlying mechanisms by which aquatic exercises influence these malalignments could lead to more effective interventions. This study aimed to evaluate the effects of aquatic exercises on ankle-foot malalignments in obese postmenopausal women. Additionally, it aimed to compare the outcomes of aquatic exercise programs with those of land-based exercises in terms of resistance training, balance, and proprioception. By addressing this gap, the study aspires to provide valuable insights for developing targeted rehabilitation strategies for this underserved population.

## Materials and methods

**Figure 1:**
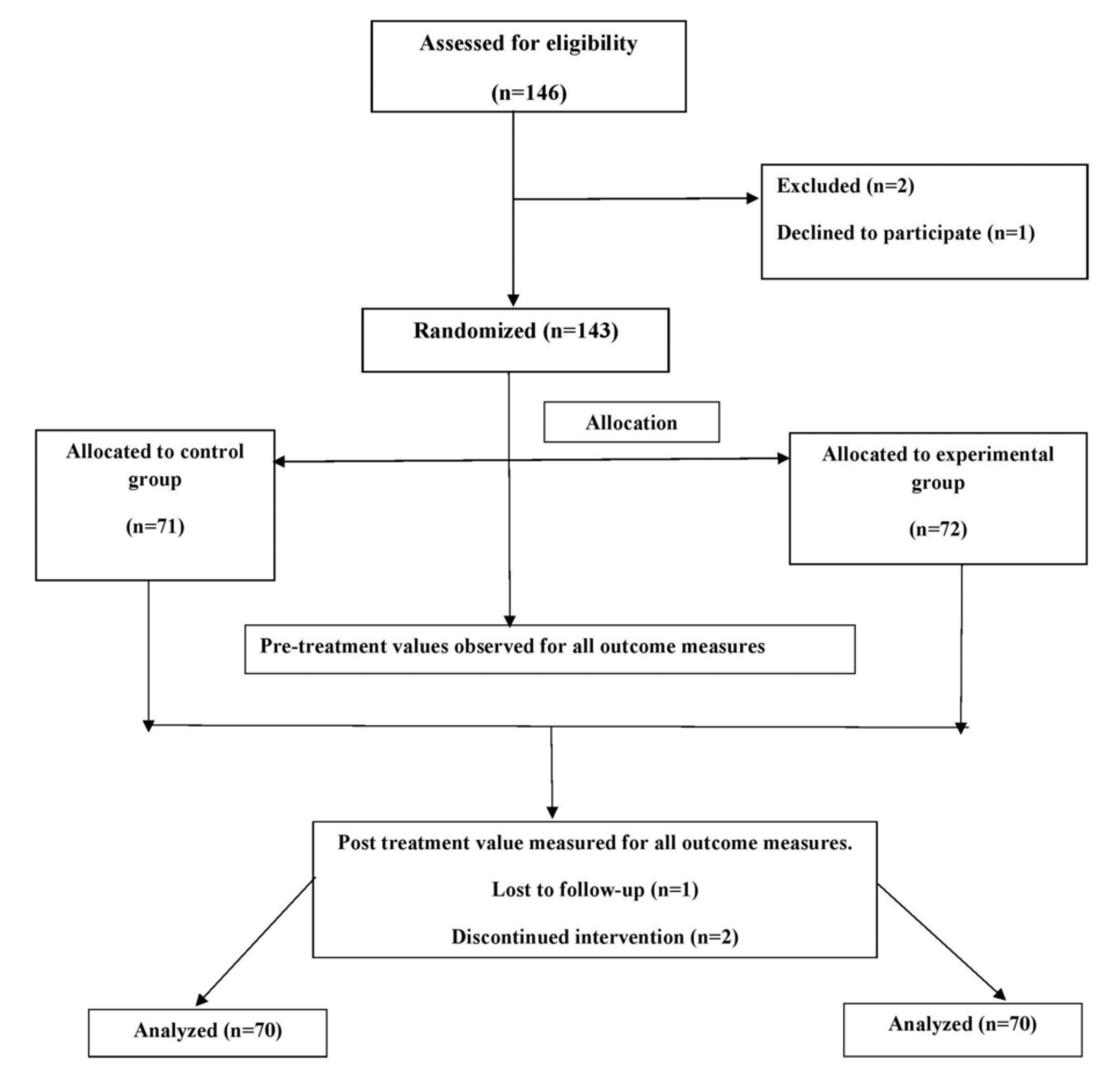
CONSORT flowchart CONSORT: Consolidated Standards of Reporting Trials

The study commenced after approval from the institutional protocol and ethics committee. It was conducted with a total of 146 obese postmenopausal women, using the computerized Statistical Package for the Social Sciences (SPSS) software for data analysis. The intervention has been described in accordance with the Template for Intervention Description and Replication checklist, which is provided in the Appendix. The study included obese women, with a body mass index (BMI) of 30-39.9 kg/m², covering Class I (30.0-34.9) and Class II (35.0-39.9) obesity. Women with a BMI of ≥40 (Class III) were excluded [5]. Participants with a BMI lower than 30 kg/m² (i.e., normal weight and overweight categories) and those who were uncomfortable with aquatic-based activities (hydrophobic) were excluded from the study. Additionally, individuals with open wounds, recent fractures, or skin infections were not included. Women diagnosed with neurological or respiratory disorders, as well as those with psychiatric conditions, were also excluded to ensure the safety and integrity of the results. The participants were randomly assigned to two groups: Group A, which underwent land-based exercises, and Group B, which participated in aquatic exercises (Figure 1). Randomization was carried out based on the established inclusion and exclusion criteria and by giving them a sealed envelope to categorize the individuals in the two groups by randomized allocation sequence [12]. Before starting the study, all participants received a detailed explanation of the research procedures and objectives. They were then asked to provide written informed consent, confirming their voluntary participation and understanding of the study protocol.

Assessment for malalignment

Foot Posture Index-6

The Foot Posture Index is a reliable and comprehensive tool used to assess foot posture, analyzing six specific parameters to evaluate alignment and biomechanics. It demonstrates high intrarater reliability (intraclass correlation coefficient, ICC: 0.96, p < 0.001), ensuring consistent measurements. The assessment includes palpation of the talar head, examination of supra- and inframalleolar curvatures, talonavicular prominence, and calcaneal frontal plane position. Additionally, it evaluates forefoot abduction/adduction relative to the rearfoot and the medial longitudinal arch's congruence [13,14].

Navicular Drop Test

The navicular drop test assesses foot pronation by measuring the shift in the navicular bone’s position. The examiner starts by palpating the navicular bone and measuring the vertical distance from it to the ground while the participant is seated and non-weight-bearing. This serves as the baseline. The measurement is then repeated with the participant standing, placing the foot in a weight-bearing position. The difference between the seated and standing measurements is calculated, known as the navicular drop. A navicular drop greater than 1 cm indicates excessive pronation, which suggests a potential issue with foot biomechanics [15].

Medial Longitudinal Arch Angle

The medial longitudinal arch angle (MLAA) is a widely used method for assessing foot posture. It is measured by identifying and marking three key anatomical landmarks: the midpoint of the medial malleolus, the most prominent point of the navicular tuberosity, and the most medial prominence of the first metatarsal head. These points are located through palpation and marked on the skin with a marker pen. A goniometer is then used to measure the angle by aligning its center with the navicular mark and its arms connecting the navicular point to the medial malleolus and the first metatarsal head. The resulting obtuse angle is recorded as the MLAA. Based on the measurement, angles less than 130° indicate a pronated foot type, angles between 130° and 150° represent a neutral foot type, and angles greater than 150° suggest a supinated foot type [16].

Treatment protocol

All the participants, before filling out the consent form, were briefed on the purpose and methodology of the study. In this research, 146 postmenopausal obese women were selected, whose musculoskeletal assessment was taken to confirm malalignments among them. She had been put on the treatment for six weeks with four sessions a week. One session took around 45 minutes. The participants were categorized into two groups, with 73 participants in each group, through a sealed envelope to categorize the individuals in the two groups by a randomized allocation sequence. Group A performed land-based exercises, whereas Group B exhibited Aquatic exercises. Group A exhibited the exercises in the Outpatient Department. Group B performed the exercises in a hydrotherapy pool with a temperature of 26°C-36°C, which is adequate for an individual. Exercises were conducted under the supervision of a registered physiotherapist. A standardized exercise protocol had been given to both groups (Table 1).

**Table 1:** Treatment protocol

Type of exercises	Land-based exercises	Repetitions/sets (weeks 1-6)	Water-based exercises	Repetitions/sets (weeks 1-6)
Warm up (10 minutes)	Walking forward, backward, and sidewalk; leg swings; and on-spot marching	10 reps × 3 sets	Walking forward, backward, and sidewalk; leg swings with pool side bar; on-spot marching	10 reps × 3 sets
Resistance/strength training	Knee flexion-extension (in sitting and standing); ankle orsiflexion and plantarflexion (sitting); forward lunges; knee raise to kick back	10 reps × 3 sets (use of low-resistance TheraBands)	Knee flexion-extension (in sitting and standing); ankle dorsiflexion and plantarflexion (sitting); forward lunges; knee raise to kick back	10 reps × 3 sets (use of aquatic cuffs)
Balance and proprioceptive training	Tandem walking for 2 m standing on Diana ball; walking on toes; multiple changes in direction drill (forward, backwards, sideways); standing clock drill; walking in a figure of 8 holding wand and then without wand; single-leg stance with and without wand; single-leg stance on hard surface and throwing the ball; double-leg stance with arm abducting and holding a weight	10 reps × 3 sets	Tandem walking for 2 m; standing on wobble disc; walking on toes; multiple changes in direction drill (forward, backwards, and sideways); standing clock drill; walking in a figure of 8 holding noodle and then without noodle; single-leg stance with and without noodle; single leg stance on hard surface and throwing ball; double-leg stance with arm abducting and holding a weight	10 reps × 3 sets (with aquatic equipment)
Cool down (10 minutes)	Lying on the floor stretching: plantaris, Achilles, and gastrosoleus	4-5 minutes, 30 seconds × 3 sets	Supported cycling against the wall; stretching: Achilles, plantaris, and soleus	4-5 minutes, 30 seconds × 3 sets

**Figure 2:**
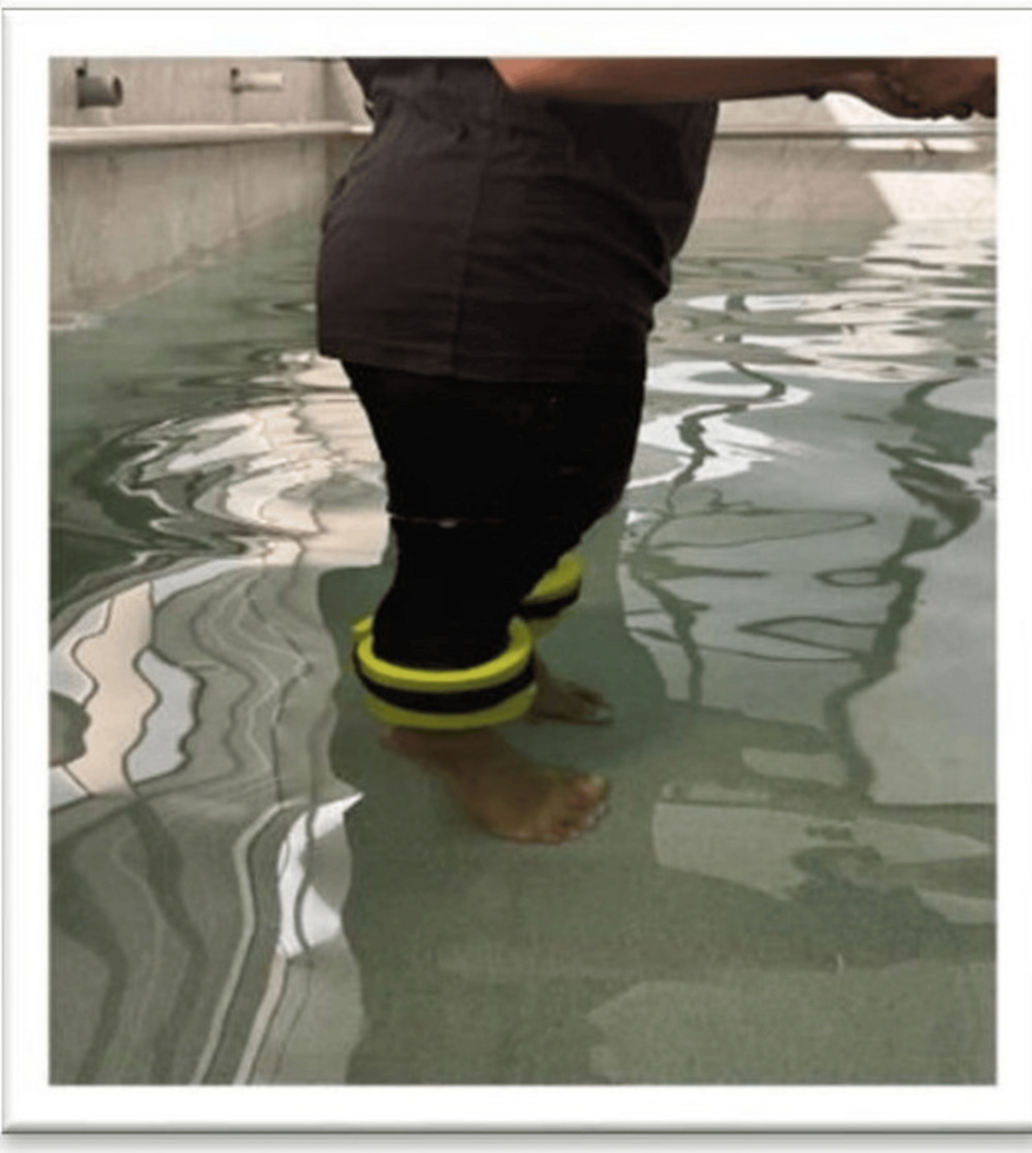
Heel raise with aquatic weight cuff

In Figure 2, the patient is seen doing a heel raise exercise with the aquatic weight cuff.

**Figure 3:**
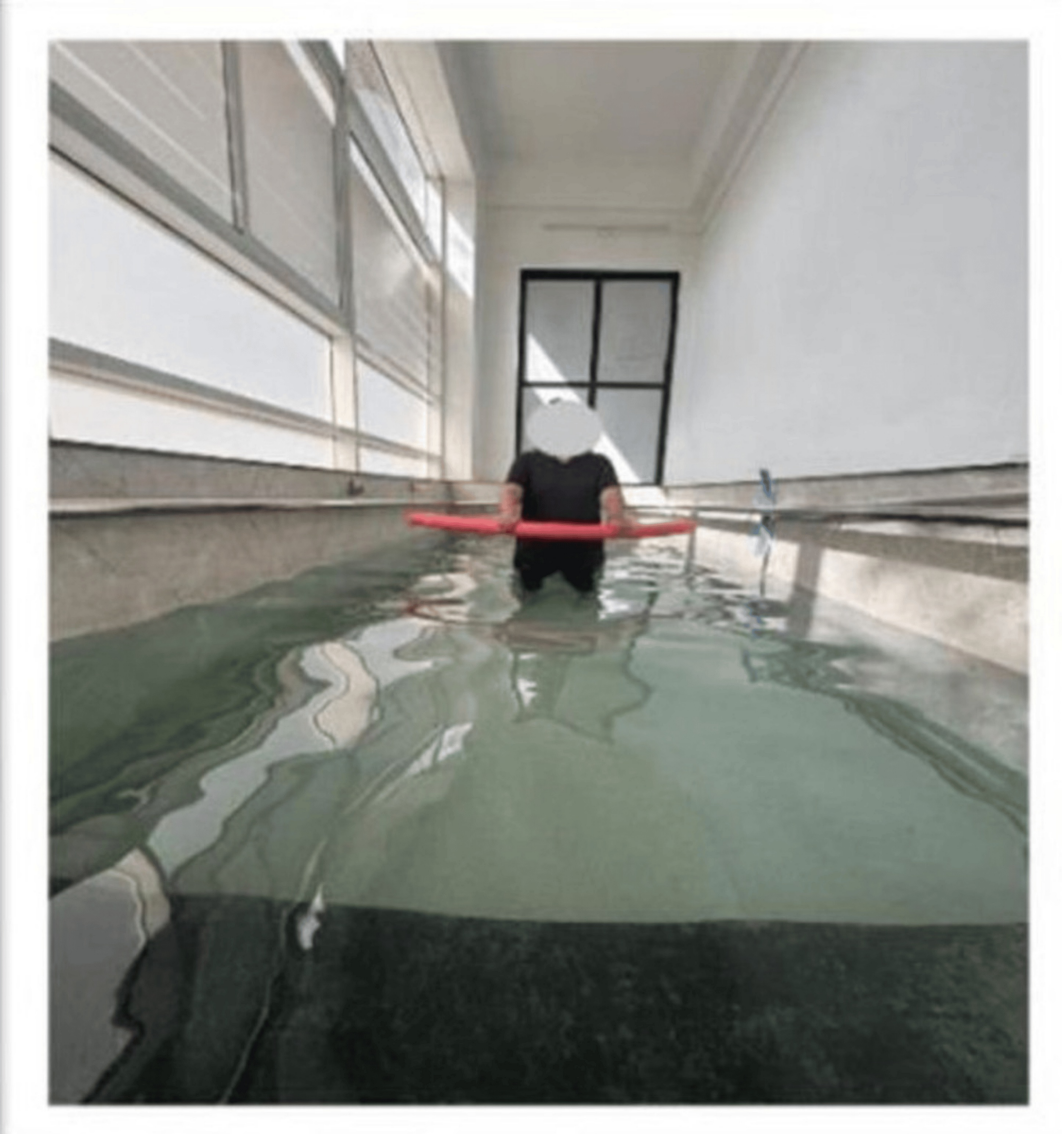
Walking with an aquatic noodle

In Figure 3, the patient is seen walking with the aquatic noodle.

Outcome measures

Numerical Pain Rating Scale

The Numeric Pain Rating Scale is a widely used tool for assessing pain intensity. It is an 11-point scale with numbers ranging from 0 to 10, designed to capture the subjective experience of pain. A score of 0 indicates "no pain," representing an absence of any discomfort. In contrast, a score of 10 denotes the "worst imaginable pain," signifying the most severe level of pain a person can conceive. Patients are guided to select a single number from the scale that they feel most accurately reflects the intensity of their current pain [17].

Manual Muscle Testing

MMT is a clinical technique used to assess the functionality and strength of specific muscles or muscle groups. This procedure involves analyzing how well a muscle performs a particular movement when subjected to the opposing forces of gravity and external manual resistance. When performing strength testing, the targeted muscle or muscle group is first isolated to ensure an accurate evaluation. External force is then applied to assess the muscle's response, using one of two primary methods: the break test or the make test. In the break test, resistance is applied at the end of the muscle's tested range of motion while the individual attempts to maintain the position. This method typically results in higher strength grades because it evaluates the muscle at its peak isometric capacity. Conversely, in the make test, resistance is applied continuously throughout the muscle's range of motion as the individual actively performs the movement [18].

Star Excursion Balance Test

The Star Excursion Balance Test (SEBT) is a reliable and valid tool for assessing dynamic postural control of the lower limb. Initially, the SEBT involved standing at the center of an eight-pointed star, with lines extending in eight directions, each separated by 45°. During the test, participants stand barefoot in a double-limb stance (feet together) at the center of the grid. They then reach as far as possible along each direction with the furthest part of their reaching foot, touch the line, and return to the starting position while maintaining balance. The trial ends when the participant regains their initial double-limb stance. The SEBT demonstrates strong reliability, with intrarater ICC values ranging from 0.85 to 0.91 and interrater ICC values ranging from 0.99 to 1 [19-21].

Statistical analysis

Both manual analysis and software analysis using SPSS version 26.0 (IBM Inc., Armonk, NY) were carried out. The numerical data were presented as means and standard deviations, and a paired t-test was employed to compare pre- and post-intervention data within the group. Continuous variables underwent normality testing. To assess significant changes over time, a repeated-measures analysis was conducted. A significance criterion of p < 0.0001 was set for all analyses to ensure robust statistical findings. This methodology provided a thorough evaluation of the data, integrating manual checks with advanced software analysis.

## Results

Table 2 illustrates the demographic variability observed in this study, highlighting the characteristics of participants from both the land-based therapy group and the aquatic-based therapy group. The data indicate that the greater number of participants belong to the 50-55 years age group, which aligns with the typical age range of postmenopausal women, along with the mean BMI among all the participants.

**Table 2:** Demographic variables BMI: body mass index

Variables	Group A	Group B
Age		
45-50 years	21 (48.83%)	22 (51.16%)
50-55 years	22 (51.16%)	21 (48.83%)
55-60 years	27 (50%)	27 (50%)
BMI (kg/m^2^)	33.76 ± 2.3	35.65 ± 4.6

Table 3 details the Numerical Pain Rating Scale (NPRS) results, comparing pain levels in both groups before and after the intervention. The findings reveal that Group B, which participated in aquatic-based therapy, experienced significantly greater pain reduction both at rest and during activity compared to Group A, which underwent land-based therapy. The effect size for Group B was large for both conditions, with Cohen’s d = 5.72 (95% confidence interval, CI: 5.18-6.26) during activity and 1.76 (95% CI: 1.41-2.11) at rest, indicating a substantial treatment impact. In contrast, Group A showed only small to moderate effect sizes: Cohen’s d = 0.38 (95% CI: 0.13-0.62) on activity and 0.43 (95% CI: 0.17-0.69) at rest. This suggests the superior efficacy of aquatic therapy in managing pain.

**Table 3:** Numerical Pain Rating Scale

NPRS	Group A		Mean difference	p value	t value	Group B		Mean difference	p value	t value
	Pre	Post				Pre	Post			
On activity	6.7 ± 1.33	6.2± 1.32	0.5	0.0029	3.088	7.5 ± 0.9	2.35 ± 0.9	5.15	<0.0001	39.757
At rest	3.54 ± 1.48	3.01± 0.789	0.53	0.0071	2.773	3.54 ± 01.48	1.61 ± 0.6	1.93		10.383

Table 4 showcases the muscle strength of the ankle and foot complex movements, including dorsiflexion, plantarflexion, eversion, and inversion, as measured through MMT before and after the intervention. The data demonstrate that the aquatic-based therapy group achieved superior outcomes in improving muscle strength across all movements compared to the land-based group. The effect sizes in the aquatic group were large for all parameters, with Cohen’s d ranging from 0.94 to 2.24, and the 95% CIs indicating consistently strong improvements (e.g., inversion: d = 0.94, 95% CI: 0.61-1.27; plantarflexion: d = 2.24, 95% CI: 1.85-2.63). In contrast, the land-based group showed only small to moderate improvements (Cohen’s d ranging from 0.37 to 0.51). Notably, the greatest improvement in the aquatic group was observed in plantarflexion strength, followed by dorsiflexion, inversion, and eversion, reinforcing the enhanced efficacy of aquatic therapy in strengthening the ankle-foot complex.

**Table 4:** Manual muscle testing (within group analysis) This table presents pre- and postintervention scores for ankle-foot complex muscle strength (plantarflexion, dorsiflexion, eversion, and inversion) within Group A (land-based) and Group B (aquatic-based) ^*^Statistically significant difference

MMT	Group A		Mean difference	t value	p value	Group B		Mean difference	t value	p value
	Pre	Post				Pre	Post			
Plantarflexion	2.81 ± 0.72	3.11 ± 0.89	0.30	0.0284	2.239	2.6 ± 0.69	4.04 ± 0.57	1.44	13.439	<0.0001^*^
Dorsiflexion	2.54 ± 0.7	2.9 ± 0.7	0.36	2.965	0.0042	2.68 ± 0.5	3.5 ± 0.79	0.82	9.623	<0.0001^*^
Eversion	2.42 ± 0.67	2.7 ± 0.7	0.28	2.786	0.0069	2.58 ± 0.6	3.28 ± 0.78	0.70	6.048	<0.0001^*^
Inversion	2.78 ± 0.8	3.05 ± 0.6	0.27	2.325	0.0230	2.78 ± 0.88	3.61 ± 0.92	0.83	6.285	<0.0001^*^

Table 5 displays the balance and proprioception outcomes, measured using the SEBT. The results indicate a clear advantage for the aquatic-based therapy group, which demonstrated significantly greater postintervention improvements across all directions, particularly in the posterolateral (PL) direction. The effect sizes were notably large in the aquatic group, with Cohen’s d = 2.62 (95% CI: 2.19-3.05) for the anterior, 3.77 (95% CI: 3.27-4.26) for the PL, and 3.18 (95% CI: 2.71-3.65) for the posteromedial direction, indicating a strong impact on dynamic balance and proprioception. In contrast, the land-based group showed only small to moderate improvements, with effect sizes ranging from 0.34 to 0.52. These findings suggest that aquatic therapy is more effective in enhancing balance and proprioception compared to land-based therapy, and they further highlight the efficacy of the SEBT in detecting and tracking improvements in these domains.

**Table 5:** Star Excursion Balance Test (within group analysis) This table displays pre- and postintervention results of the Star Excursion Balance Test in the anterior, posterolateral, and posteromedial directions within Groups A and B

Direction	Group A		Mean difference	t value	p value	Group B		Mean difference	t value	p value
	Pre	Post				Pre	Post			
Anterior	61.25 ± 5.27	64.82 ± 7.67	3.57	3.036	0.0034	60.58 ± 3.85	73.24 ± 5.66	12.66	14.939	<0.0001
Posterolateral	57.82 ± 7.4	62.07 ± 14.97	4.25	3.034	0.0034	64.90 ± 3.166	90.24 ± 7.76	25.34	25.278	<0.0001
Posteromedial	62.85 ± 7.96	67.44 ± 18.59	4.59	2.230	0.0290	68.76 ± 3.8	89.97 ± 6.6	21.21	22.459	<0.0001

Table 6 states the posttest assessment values between the groups. Group B demonstrated significantly better outcomes compared to Group A across all measures. In the NPRS, pain on activity was significantly lower in Group A (p < 0.0001), while pain at rest also showed a notable difference. MMT revealed significant improvements in Group B for plantarflexion (p = 0.0004), eversion (p < 0.0001), and other movements. The SEBT showed marked improvements in Group B, particularly in posterolateral reach (p < 0.0001). These results indicate that Group B had superior pain relief, muscle strength, and balance performance, suggesting a more effective intervention.

**Table 6:** Group analysis (between-group comparison) This table compares postintervention scores between Groups A and B across multiple outcome measures, including pain intensity (NPRS), muscle strength (MMT), and dynamic balance (SEBT) NPRS: Numerical Pain Rating Scale; MMT: manual muscle testing; SEBT: Star Excursion Balance Test

Outcome measures		Post (group A)	Post (group B)	p value	t value
NPRS	On activity	6.21 ± 1.32	2.35 ± 0.90	<0.0001	20.2
	At rest	3.01 ± 0.78	1.61 ± 0.64		11.501
Manual muscle testing	Plantarflexion	3.1 ± 0.8	3.7 ± 1.02	0.0004	3.872
	Dorsiflexion	2.9 ± 0.7	3.5 ± 0.7	<0.0001	4.502
	Eversion	2.7 ± 0.74	3.28 ± 0.78		4.523
	Inversion	3.05 ± 0.6	3.61 ± 0.9		4.073
Star Excursion Balance Test	Anterior	64.82 ± 7.6	73.24 ± 5.6	<0.0001	7.382
	Posterolateral	62.07 ± 14.97	90.24 ± 7.76		13.974
	Posteromedial	67.44 ± 18.59	89.97 ± 6.65		9.485

## Discussion

Postmenopausal women undergo physiological changes that include decreased bone density, hormonal changes, and altered musculoskeletal function, which predispose them to biomechanical issues such as ankle-foot malalignments. Malalignments are improper positioning of the ankle and foot, which can impair gait, increase pain, and elevate the risk of falls. Obesity in this demographic exacerbates these challenges, as excess weight places excessive strain on the lower extremities, further compromising alignment and stability. The results of the present study clearly indicate that aquatic-based resistance, balance, and proprioceptive training produced superior therapeutic outcomes compared to land-based interventions in obese postmenopausal women with ankle-foot malalignments. Participants in the aquatic therapy group demonstrated significantly greater reductions in pain levels, both at rest and during activity, as assessed by the NPRS. This can be attributed to the unique properties of water, such as buoyancy and hydrostatic pressure, which reduce joint compression and allow movement with less discomfort, facilitating early pain-free mobility. In terms of muscle strength, MMT revealed marked improvements in all ankle-foot muscle groups, plantarflexors, dorsiflexors, invertors, and evertors, following aquatic training. The resistance offered by water during dynamic movements likely provided continuous yet safe overload, contributing to enhanced muscular activation and endurance. Furthermore, the SEBT scores significantly improved in all directions, particularly PL and posteromedial, reflecting notable gains in dynamic balance and proprioception. The structured protocol, which included a combination of warm-up, resistance, balance, and cool-down phases over six weeks, appeared to engage multiple systems, muscular, neurological, and sensory, in an integrative manner. Overall, the study reinforces the role of aquatic therapy as a multidimensional intervention capable of reducing pain, strengthening key muscle groups, and restoring functional balance in this high-risk population.

The study by Sadaak et al. concluded that aquatic therapy was more effective than traditional physiotherapy in reducing pain and restoring dynamic balance in elite athletes with Grade III ankle sprains. Although their population differed from ours, the similarity in outcome, namely significant improvements in pain relief and balance, supports our findings that aquatic-based training effectively enhances neuromuscular performance and functional mobility. While their focus was on acute injuries in athletes, our study extended these benefits to a chronic condition in postmenopausal obese women, indicating the broader applicability of aquatic interventions across different populations and clinical conditions [22].

Research conducted by Ansari et al. provided valuable insights into the benefits of aquatic training. Their study disclosed that after eight weeks of structured aquatic exercise, participants exhibited a significant increase in muscle electromyography (EMG) values. This finding highlights the potential of aquatic training in improving neuromuscular activity and muscle performance. Building on this foundation, the present study set out to investigate the broader implications of eight weeks of aquatic exercise training, particularly focusing on its effects on functional disability, flexibility, and muscle EMG values. The population that was chosen for this particular research was postmenopausal women with chronic low back pain. However, unlike Ansari et al.’s research, which primarily examined neuromuscular activity using EMG to compare land-based and aquatic resistance training, our study delves deeper into additional variables. Specifically, we addressed the unique needs of postmenopausal women in the obese category. To comprehensively evaluate the impact of aquatic training, our research incorporated elements of resistance training, balance improvement, and proprioceptive enhancement. Another significant distinction in our methodology was the choice of evaluation techniques. While the study by Ansari et al. relied on EMG measurements to assess muscle activity, our research employed MMT. This hands-on approach involves evaluating muscle strength through manual resistance, offering a practical and reliable alternative to instrument-based assessments [23].

A study conducted by Jain and Shinde investigated the effects of an eight-week aquatic training program on individuals suffering from bilateral KOA. The findings reflected that this program effectively reduced pain levels and enhanced lower limb muscle performance in the patients. Both groups in the study exhibited substantial improvements in their Visual Analog Scale and Western Ontario and McMaster Universities Osteoarthritis Index scores, reflecting reduced pain and better joint functionality. However, Group B outperformed Group A in various key performance metrics, including the one-repetition maximum leg press test, proprioception accuracy, Timed Up and Go test, and the 40-m fast-paced walk test. In our study, it was concluded that water-based therapy was very effective in improving strength, balance, and proprioception in individuals with malalignments pertaining to the ankle and foot complex. This study was executed with the assistance of the MMT, SEBT, and NPRS. This study concludes that aquatic-based training yields better results in improving the pain experience compared to land-based exercises [11].

Aphale et al. evaluated the effectiveness of an aquatic exercise program in improving pain and functional performance in overweight adolescent runners with functional flat feet in their study. The results showed that aquatic exercises led to significantly greater improvements in pain reduction, foot posture, and sprint performance compared to land-based exercises. In contrast, our study examines the effects of aquatic resistance, balance, and proprioceptive training on lower extremity strength and alignment in obese postmenopausal women. The results indicate that aquatic exercises significantly improved foot posture, balance, and muscle strength compared to land-based exercises, with highly significant statistical outcomes. The findings emphasize the effectiveness of water-based training in reducing pain and enhancing postural control in this population [24].

Joshi et al. concluded that patellofemoral joint dysfunction in middle-aged women with obesity has great results with aquatic exercises as a valuable therapeutic strategy, contributing to holistic improvements in musculoskeletal well-being. It underscores the capacity of aquatic interventions to enhance the overall quality of life of individuals with patellofemoral joint dysfunction. In our study, we focused on ankle-foot alignment in obese postmenopausal women. Both studies demonstrated that aquatic exercises led to significantly greater improvements in pain reduction and functional outcomes compared to land-based exercises, emphasizing the role of water-based interventions in managing musculoskeletal conditions associated with obesity [25].

Strengths

This study employed a structured and comprehensive intervention protocol combining resistance, balance, and proprioceptive training, specifically tailored for obese postmenopausal women, a population often underrepresented in rehabilitation research. The use of both aquatic- and land-based exercise groups allowed for a comparative evaluation of intervention efficacy in different environments. Second, the study utilized validated and widely accepted outcome measures, NPRS, MMT, and SEBT, which strengthened the reliability and clinical relevance of the results. The consistent supervision of all sessions by registered physiotherapists ensured adherence to the protocol and minimized variability in intervention delivery. Additionally, the randomized allocation and clearly defined inclusion/exclusion criteria helped enhance internal validity and reduce selection bias. Collectively, these factors contribute to the robustness and applicability of the study findings in clinical practice.

Limitations

Further research could be conducted by including individuals with lower limb misalignments, such as valgus or varus deformities, to obtain a greater knowledge of how these structural variations in the ankle and foot influence the outcomes of rehabilitation programs. Incorporating participants with such conditions could enable valuable insights into the interconnected biomechanical compensations and functional limitations that may arise. Additionally, implementing a longer duration training protocol would allow researchers to observe the progressive effects of rehabilitation on recovery biomechanics over time. This extended timeline could shed light on how the intervention impacts other dimensions of recovery, such as joint stability, balance, and gait patterns, as well as the overall quality of life.

## Conclusions

The study concluded that a six-week aquatic training program effectively reduced pain and enhanced the overall performance of the ankle-foot complex in obese postmenopausal women. Improvements were observed in strength, balance, and proprioception, which collectively contributed to a better quality of life for the participants. Furthermore, this intervention demonstrated potential in preventing the progression of additional musculoskeletal issues commonly experienced by obese postmenopausal women, especially in the distal most part of the lower limb.

## Appendices

Template for intervention description and replication checklist

1. Brief name

Aquatic-based resistance, balance, and proprioceptive training program for postmenopausal obese women with ankle-foot malalignments.

2. Why (rationale)

The intervention aims to address musculoskeletal changes associated with menopause and obesity, specifically pain, postural imbalance, and muscle weakness leading to ankle-foot malalignment. Aquatic therapy was chosen for its low-impact, resistance-providing environment, which enhances safety and efficacy for this demographic.

3. What materials

a. Aquatic equipment: aquatic cuffs, noodles, wobble discs, and weighted wands. b. Land-based equipment: TheraBands (low resistance) and the Diana ball. c. Hydrotherapy pool (26°C-36°C). d. Exercise protocol chart (six-week schedule). e. Manual documentation tools: Foot Posture Index sheets, MMT forms, NPRS.

4. What procedures

The six-week program consisted of structured warm-up, resistance/strength training, balance/proprioceptive exercises, and cool-down phases. Group A received land-based exercises; Group B received the same protocol in water using aquatic aids. Sessions were supervised and included walking drills, ankle/knee movements, static and dynamic balance drills, and stretching routines.

5. Who provided

All sessions were conducted under the supervision of registered physiotherapists with expertise in musculoskeletal rehabilitation and aquatic therapy.

6. How (mode of delivery)

Face-to-face delivery in group sessions (hydrotherapy pool or OPD gym setting), conducted four times a week.

7. Where

a. Group A: Physiotherapy Outpatient Department (OPD), Krishna Institute. b. Group B: Hydrotherapy unit, Krishna Institute, with appropriate safety measures and water conditions.

8. When and how much

a. Duration: six weeks b. Frequency: four sessions per week c. Session duration: ~45 minutes Each session included warm-up, strengthening, balance/proprioceptive tasks, and cool down

9. Tailoring

The intervention followed a standardized protocol, but exercise intensity (e.g., resistance band color or aquatic equipment load) was adjusted based on the individual’s tolerance and progression.

10. Modifications

No major modifications were made to the intervention during the study.

11. How well (planned fidelity)

Adherence was monitored through physiotherapist supervision and participant attendance records. Therapists followed a standardized exercise protocol to ensure uniform delivery.

12. How well (actual fidelity)

The intervention was delivered as planned. No deviations from the protocol were recorded, and participant attendance was consistent across both groups.
